# Laboratory methods to decipher epigenetic signatures: a comparative review

**DOI:** 10.1186/s11658-021-00290-9

**Published:** 2021-11-11

**Authors:** Raheleh Halabian, Valizadeh Arshad, Ali Ahmadi, Pardis Saeedi, Sadegh Azimzadeh Jamalkandi, Mohammad Reza Alivand

**Affiliations:** 1 Molecular Biology Research Center, Systems Biology and Poisonings Institute, Baqiyatallah University of Medical Sciences, Tehran, Iran; 2 Department of Stem Cell and Developmental Biology, Cell Science Research Center, Royan Institute For Stem Cell Biology and Technology, ACECR, Tehran, Iran; 3 Applied Microbiology Research Center, Systems Biology and Poisonings Institute, Baqiyatallah University of Medical Sciences, Tehran, Iran; 4 Chemical Injuries Research Center, Systems Biology and Poisonings Institute, Baqiyatallah University of Medical Sciences, Mollasadra Ave., 14359-16471 Tehran, Iran; 5 Department of Medical Genetics, Faculty of Medicine, Tabriz University of Medical Sciences, Tabriz, Iran

**Keywords:** Epigenetics, DNA methylation, Histone modifications, Techniques

## Abstract

Epigenetics refers to nucleotide sequence-independent events, and heritable changes, including DNA methylation and histone modification (as the two main processes), contributing to the phenotypic features of the cell. Both genetics and epigenetics contribute to determining the outcome of regulatory gene expression systems. Indeed, the flexibility of epigenetic effects and stability of genetic coding lead to gene regulation complexity in response signals. Since some epigenetic changes are significant in abnormalities such as cancers and neurodegenerative diseases, the initial changes, dynamic and reversible properties, and diagnostic potential of epigenomic phenomena are subject to epigenome-wide association studies (EWAS) for therapeutic aims. Based on recent studies, methodological developments are necessary to improve epigenetic research. As a result, several methods have been developed to explore epigenetic alterations at low, medium, and high scales, focusing on DNA methylation and histone modification detection. In this research field, bisulfite-, enzyme sensitivity- and antibody specificity-based techniques are used for DNA methylation, whereas histone modifications are gained based on antibody recognition. This review provides a mechanism-based understanding and comparative overview of the most common techniques for detecting the status of epigenetic effects, including DNA methylation and histone modifications, for applicable approaches from low- to high-throughput scales.

## Introduction

The term “epigenetics” previously referred to indefinite genetic principles first introduced by Conrad Waddington [1, 2], but now, epigenetics refers to non-genetic heritable genomic modifications involved in gene expression regulation without any changes in the DNA sequence [3, 4]. The importance of epigenetics has now been realized at the molecular level in vital processes such as cell destiny, self-recognition, phenotypic plasticity, evolution, and ecology. Therefore, the unique memory of the epigenetic landscape is a bridge between the genotype and phenotype of differentiated cells and organs [5, 6].

The integrated interplay between the genetic and epigenetic coding determines the final transfer of regulatory signals. The epigenetic flexibility and genetic coding stability provide a complex pattern of gene expression regulation in response to internal and external stimuli. Mainly, two widely studied mechanisms of epigenetic changes in mammals are DNA methylation and histone modification, and both can find a reading code by effector proteins to modulate transcription [7–9].

Disruptions of DNA methylation arise from malfunctions in activity of writer, eraser, and reader proteins [10]. DNA methyl transferases (*DNMT1* and *DNMT3a/3b*), as a writer, add just a methyl group to the CpG dinucleotide (on islands or non-islands), and CpG from an S-adenosyl methionine (SAM) source [2]. In contrast, ten-eleven translocations *TET-1*, *TET-2*, and *TET-3*, as oxygenases, remove a methyl group during a three-step base modification mechanism including 5-hydroxymethyl cytosine (5hmC), 5-formyl cytosine (5fC), and 5-carboxyl cytosine (5caC), respectively. Based on the evidence, all modified cytidines are important as epigenetic signatures to promote cell development and differentiation pathways. According to the role of these elements in cell and organ health, the detection of these modifications, especially for 5mC, is essential and completely applicable for diagnosing abnormalities [9–12].

Histone modification is another part of the epigenetics phenomenon, which relates the environment and genetics in the gene expression process. Histones (H3, H4, H2A, H2B, and H1) are conserved core proteins involved in DNA packaging. Histone proteins are affected by post-translational modifications, including methylation, acetylation, ubiquitination, and SUMOylation on lysine residues. Many histone modifications mediate significant functional changes in chromatin structures. They may affect either the nucleosome structure or the dynamic compacting of nucleosomes. Besides packaging, chromatin structure modulates gene expression according to the histones’ post-translational modifications. These modifications are site-specific and can dramatically change many biological processes [13]. The disruptive changes in epigenetic factors may cause diseases. Also, genetic mutations, in epigenetic modifiers that cause different diseases, affect chromatin either in trans or cis in changing chromatin structure [2, 3]. To investigate substantial epigenetic markers and alterations, several low and high throughput methods with different specificities and sensitivities have been developed with direct and indirect assays [14–16]. In this review, we aimed to provide a comprehensive report with a panel of methods that can be applied to research in this field. Methods included in the analysis of methylation modifications, based on restriction enzyme digestion, bisulfite, and affinity enrichment, may assist further techniques such as PCR and microarray. Then, there will be an overview of histone modification analysis, discussing several techniques, such as the chromatin immunoprecipitation assay, modified ChIP methods, site-specific analysis of histone modifications, and DNase I hypersensitivity analysis of chromatin structure, along with the high throughput next-generation sequencing-based method.

## Overview of DNA methylation analysis

DNA methylation, at the nucleotide level, is a fundamental epigenetic signature. There is various evidence concerning the substantial role of the methyl groups in the genome in control of gene expression. In most complex diseases, such as cancers and neurodegenerative diseases, an aberrant methylation profile occurs in DNA [17–19].

There are three ways for writing, erasing, and reading the methyl group in CpG. DNMT1 and DNMT3a/3b add a methyl group to cytosine by SAM substrate. Also, DNMT1 acts on hemimethylated DNA and maintains methylation patterns after DNA replication, where DNMT3a/3b performs a de novo methylation [10, 11, 20]. Meanwhile, TET family (TET-1, -2, -3) enzymes, as oxygenases, remove a methyl group during the conversion of 5-hydroxymethylcytosine (5hmC), 5-formyl cytosine (5fmC), and 5-carboxyl methylcytosine (5CamC). The third factor is methyl binding domain proteins (MBDs), acting as readers, which recognize and bind to methylated CpG and translate their modifications via interacting with other proteins such as transcription factors and splicing-related proteins [12].

To detect DNA methylation, traditional molecular techniques, such as PCR or cloning methods, are not applicable, because methyl groups are not copied during PCR amplification [21]. So, for DNA methylation detection, all related detection methods are required to perform a pretreatment process on the original and intact methylated DNA strand to discriminate methylation from non-methylation regions on a specific region or on the genome-wide scale [11, 22].

Dynamic modifications in DNA methylation result in various phenotypic differences in different organisms. In *Homo sapiens*, the methylation pattern of “5-methylcytosine” in the CpG dinucleotides changes the gene regulation during differentiation, genome imprinting, and X chromosome inactivation. Increased DNA methylation density has inhibitory effects by recruiting the methyl-binding proteins, transcription factor inhibition, and blocking the regulatory regions and chromatin remodeling. Dynamic alterations in the density of genomic methylation patterns can lead to dynamic changes in gene expression in response to various internal or external factors. Both normal and abnormal methylation patterns induce various diseases such as cancer. The accuracy, sensitivity, speed, simplicity, and cost of methods for methylation assessments are very diverse. Therefore, selecting the appropriate strategy requires essential considerations. Here, an overall outlook provides methods based on the advantages and disadvantages of choosing the best application method. Accordingly, investigations of genome-wide (whole genome) and targeted methylation are important for understanding the role of normal and abnormal epigenetic modifications [10, 20, 23]. The most common technique, based on bisulfite treatment, is used with PCR, sequencing, or other methods. Also, methylation-sensitive restriction enzyme-based methods are the first ones associated with other related procedures such as hybridization and PCR.

Moreover, the affinity capture of the methyl group through antibody or methyl binding proteins is another strategy. Since all the modified techniques of CpG methylation require pretreatment before detection [24, 25], based on the scale of the generated data, DNA methylation techniques are classified into low, medium, and high-throughput technologies. According to previous studies, we classify these techniques based on DNA pretreatment processes into three main techniques: (a) restriction enzyme digestion-based techniques, (b) bisulfite-based techniques, and (c) affinity enrichment-based techniques.

### Restriction enzyme digestion-based techniques

Restriction endonucleases are a pioneering approach for the detection of specific nucleotides in molecular genetics, evolved by bacteria to protect their genome from epigenetic modifications [1]. Some endonucleases are sensitive to modifications (e.g., methylation) in the CpG context of digested sequences, including *HpaII* and *SmaI*, which are the most important in DNA methylation discrimination. *HpaII* digests the unmethylated [26] CpG in the CCGG sequences, while its isoschizomeric form, *MspI*, is non-sensitive to the methylated sequences, and as a result, it digests both methylated and unmethylated sequences. The neoschizomer of *SmaI* is *XmaI*, which is not sensitive to methylation of CpG in its recognition site. Therefore, the combination of these restriction enzymes helps to detect the methylated pattern of DNA [24, 27].

Furthermore, there is an alternative endonuclease with low specificity that recognizes two sequential CpGs of the genome and called *McrBC* (*RmeC* (N)55–103 *RmeC*, R is A or G). This enzyme could identify almost all nearby methylated CpGs within the genome (40–3000 bp). There are some approaches concerning this endonuclease on regional and high throughput levels for the close region or locus-specific evaluation of methylated DNA in CpGs that will be mentioned later [15, 25, 27].

However, based on bioinformatics analysis, the coverage of methylation-sensitive restriction enzymes (MREs) such as *HpaII (*5′-CˇCGG-3′*)*, *SmaI* (5′-CCCˇGGG-3′) and two others called *NotI* (5′-GCˇGGCCGC-3′), and *BstUI* (5′-TTˇCGAA-3′), is 19% more than *McrBC* endonuclease on CpGs. The *McrBC* endonuclease is more suitable than the mentioned endonucleases for the high-throughput comparative study [16]^16^. According to various studies, the most common endonuclease-based techniques of DNA methylation assessment are listed as follows.

#### Methylation-sensitive amplified fragment length polymorphism (MS-AFLP)

**Fig. 1:**
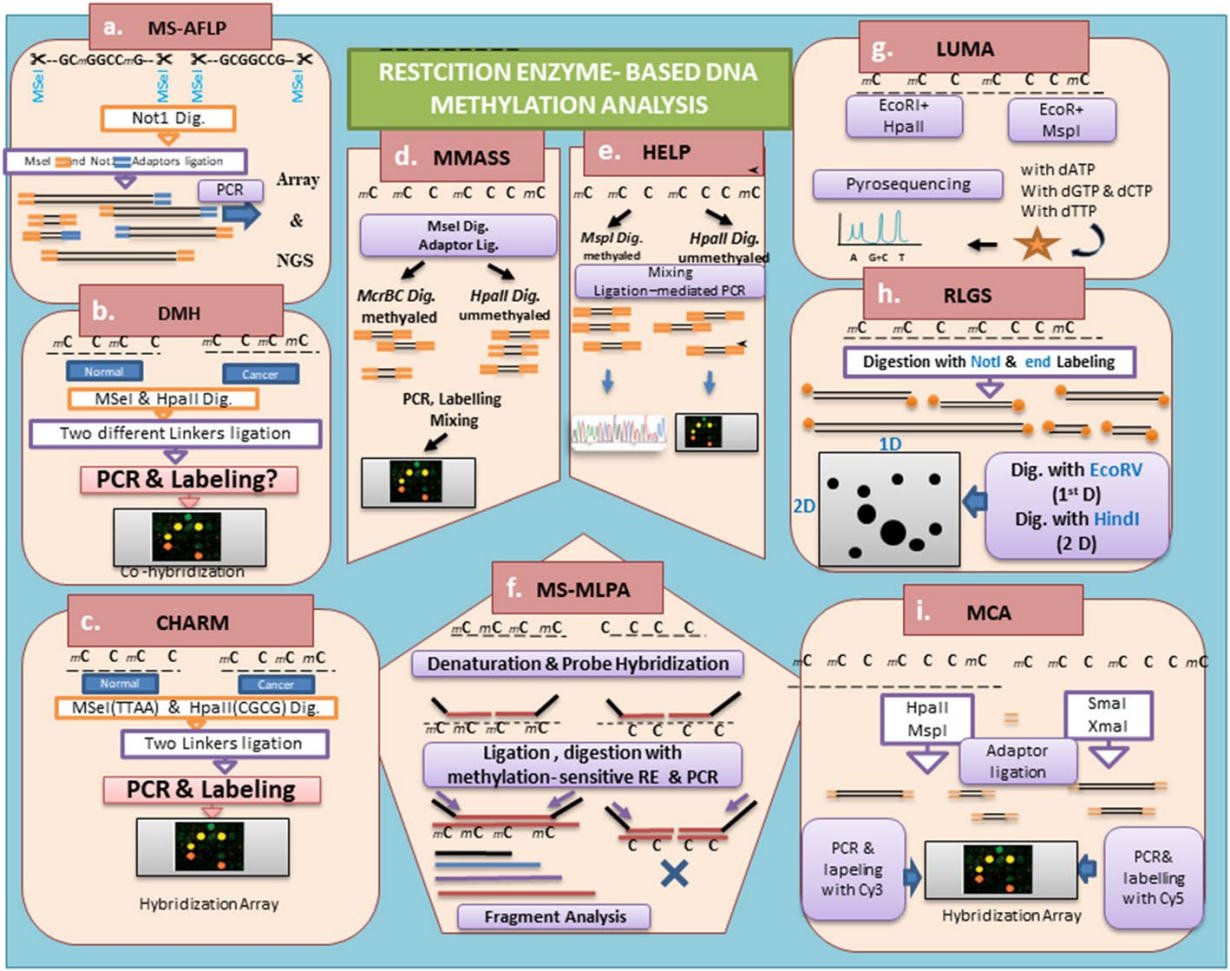
The different methods derived from restriction enzyme-based DNA methylation analysis. **a** MS-AFLP (methylation-sensitive amplified fragment length polymorphism), **b** DMH (differential methylation hybridization), **c** CHARM (comprehensive high-throughput arrays for relative methylation), **d** MMASS (microarray-based methylation assessment of single samples), **e** HELP (HpaII tiny fragment enrichment by ligation-mediated PCR), **f** MS-MLPA (methylation-specific multiplex ligation-dependent probe amplification), **g** LUMA (luminometric methylation assay), **h** RLGS (restriction landmark genomic scanning) and **i** MCA (methylated CpG island amplification)

Methylation-sensitive amplified fragment length polymorphism (MS-AFLP) is a highly sensitive and consensus method which combines two simple primary methods: methylation-sensitive restriction enzyme digestion by *Not I* and amplified fragment length polymorphism (AFLP). In this route, the genomic DNA is treated with *Mse I* in TTAA sites to shorten large fragments and then *Not I* enzyme is used to cut unmethylated GCGGCCG. Therefore, the large majority of the fragments are smaller than 1 kb. Then, two different adaptors complementary to both *NotI* and *MseI* are ligated and nick sites of adaptors are repaired [28]. Next, DNA fragments are pre-amplified and then selectively amplified by PCR using special primers. Some downstream molecular techniques were developed for DNA fingerprint of methylated DNA from a locus specific to genome-wide analysis, including gel electrophoresis-, microarray array- and next generation sequencing (NGS)-based analyses [29, 30] (Fig. 1)

#### Differential methylation hybridization (DMH)

Differential methylation hybridization (DMH) is also an important array-based method after endonuclease pre-treatment. The DMH method helps to identify the changes at the whole-genome methylation level, resulting in epigenetic alterations. In this case, restriction enzymes that do not cut at CG-rich regions such as *MseI* endonuclease digest the extracted DNA, leaving most CpG islands (CGIs) intact, then specific linkers are ligated to the digested DNA. Next, the second digestion is related to MERs to cut methylated sites of DNA such as *HepaII* and *BstuI*. PCR reaction is performed to amplify the methylated fragments, with their ligated linkers. The amplified DNA fragments are labeled by two different fluorescent stains and discriminated by hybridization array analysis. Therefore, the intensity of the fluorescent signals of the arrays determines the status of DNA methylation [24, 31, 32] (Fig. 1).

To improve the efficiency of DMH-based approaches, the *McrBC* endonuclease enzyme is applied, rather than other methylation-sensitive endonucleases. These approaches are noticeable for application of microarray-based methylation assessment of single samples (MMASS) and comprehensive high-throughput arrays for relative methylation (CHARM) followed by Sanger sequencing [24, 25].

#### Comprehensive high-throughput arrays for relative methylation (CHARM)

Although comprehensive high throughput arrays for relative methylation (CHARM) is similar to MMASS and DMH methods, it is more suitable and precise to detect methylated CpGIs on differentially methylated regions (DMRs) than the mentioned method and methylated DNA immunoprecipitation (MeDIP)-based techniques (MeDIP will be described in part 2.3.1) (Fig. 3). Irizarry et al*.* reported this method for discrimination of colon tumor methylation and margins with high resolution of CpGs. This method first utilizes McrBC, which digests sequential methylated CpGs in DNA sequences, then hybridization array is performed to detect the DNA fragments. The point is that McrBC identifies the RmC (N)55–103RmC and mostly digests the methylated DNA of CpGs. A NimbleGen HD2 microarray is a commercial form of the CHARM approach that covers almost 4.5 million CpGs. Regarding the Irizarry approach, it is reported that cells have specific DMRs and are highly conserved in human and mouse samples [33, 34] (Fig. 1).

#### Microarray-based methylation assessment of single samples (MMASS)

This is a restriction enzyme- and microarray-based method to identify the ratio of methylated to unmethylated DNA fragments in a given sequence of a single sample. This high-throughput microarray differentiation method is sensitive and dynamic as it not only detects both hyper/hypo-methylated regions but also exhibits more DNA information, for instance, the copy number differences, because it has improved the use of bioinformatic analysis to predict the combination of methylation-sensitive restriction enzymes and to apply more CpG-island probes compared with the above-mentioned methods. In this approach, to shorten the fragments of DNA, the extracted DNA is firstly digested with *MseI* (TTAA), then the relative adaptors are ligated. Next, half of the fragments are treated with *McrBC* to digest methylated sequences. The other fragments are exposed to MREs such as *HpaII* to cut unmethylated sequences [35]. Eventually, both products are separately labeled through two different fluorescent stains and the labeled amplicons are hybridized to a CpG island microarray panel [36] (Fig. 1).

#### *HpaII* tiny fragment enrichment by ligation-mediated PCR (HELP)

HpaII tiny fragment enrichment by ligation-mediated PCR (HELP) is a method based on *HpaII* endonuclease and ligation-mediated PCR techniques in array hybridization [37]. This assay allows the analysis of methylation at inter- and intragenic levels of DNA [38]. HELP can be utilized in many microarray platforms or high-throughput sequencing approaches such as NimbleGen-Roche with a high-density microarray of the oligonucleotide.

This method permits the cost-effective profiling of CpG islands of hypo-methylated DNA in the genome, compared to other methods that show hyper-methylated regions [4]. Although hypo-methylated sequences are not abundant in the genome, they are found at unique regions with important functions [39]. Two isoschizomer enzymes, HpaII and MspI, digest the genomic DNA in separate reactions, one of them discriminates the hypomethylated regions (both *MspI and HpaII*) compared with methylated regions (only *MspI*). Then the oligonucleotides as a linker are ligated and used to perform ligation-mediated PCR (LM-PCR) and to amplify the digested fragments. Following double fluorophore labeling, the amplified fragments are hybridized. The scanning and signal analysis is unique in allowing comparison of the *MspI* and *HpaII*-digested fragments [37]. *HpaII* characterizes hypomethylated loci, while *MspI* represents methylated loci as an internal control of this approach [38, 39] (Fig. 1).

#### Methylation-specific multiplex ligation-dependent probe amplification (MS-MLPA)

Multiplex ligation-dependent probe amplification (MLPA) has been introduced as a new technique for the simple and reliable detection of the copy number changes in the genome. This technology applies DNA methylation analysis called methylation-specific multiplex ligation-dependent probe amplification (MS-MLPA). It is quick, easy, and semi-quantitative with a standard control to detect alterations of methylated copy numbers of DNA in most regions with minimum initial DNA. In this technique, the methylation-sensitive restriction enzyme *HhaI* (GCGC) digests unmethylated regions after ligating the probe of interest and digested probes cannot be amplified during PCR, while methylated fragments are amplified. This technology helps to analyze the methylation status in the promoter of tumor suppressor genes (TSGs) and imprinted regions such as Prader Willi/Angelman and Beck with Wiedemann/RSS disease [40]. Here, there exist two digested and undigested samples, with evaluation through several steps, including denatured DNA hybridization and ligation with specific probes, following enzymatic digestion. Then the samples are amplified by PCR, and subsequently PCR products are separated by capillary electrophoresis. Then the results are analyzed with statistical software [40, 41] (Fig. 1).

#### Luminometric methylation assay (LUMA)

Luminometric methylation assay (LUMA) has been recently reported based on pre-treatment and pyrosequencing of MREs. As mentioned, two DNA restriction enzymes, *HpaII* and *MspI*, are sensitive and non-sensitive to methylation in CCGG, respectively. Then, pyrosequencing reactions carry out the sequencing of digested DNA fragments. The *EcoRI* enzyme, as an internal control, is first utilized in both reactions with 5ʹ-AATT overhangs. The considered light signal is the outcome with the *HpaII*/*MspI* ratio that shows unmethylated DNA status. The advantages of this approach are the high specificity and low variability that are crucial for the detection of minor methylation modifications in genome-wide analysis. Additionally, in this method, a relatively small amount of DNA is needed (< 500 ng) for evaluation. The strategy of internal control is beneficial for the estimation of the amount of inputted DNA. The high quality of inputted DNA could be affected by the digestion event and polymerase extension process of the pyrosequencing approach [42, 43].

Although methylation-sensitive restriction digestion is a simple and sensitive technique for the detection of DNA methylation status, the false-positive results are an important disadvantage, the most likely cause of which is partial DNA digestion. Furthermore, the detection of DNA methylation is indirect and relates to the digestion of DNA corresponding to nuclease activity. Additionally, the lack of internal control of endonuclease enzymes, enzyme coverage, and quality and quantity of the defined DNA is a further problem in this approach [42] (Fig. 1).

#### Restriction landmark genomic scanning (RLGS)

Restriction landmark genomic scanning (RLGS) is the earliest technology in large-scale analysis of DNA methylation profiling without genome sequence information. The analysis of methylated CpG recruits two-dimensional gel and autoradiography that display visual differences in the methylation status of genome-wide sequences. The results of restriction landmark genomic scanning (RLGS) on gel clarify both copy number and methylation status of genome CpGs. This method is done by multi-stage cleavage via different restriction enzymes [44]. As mentioned above, *Not1* is a restriction enzyme that mostly has a recognition site in the CpG island that cannot cleave methylated CpGs. After digestion with *Not1*, the digestion sites are labeled with a radioactive isotope. Then, the labeled fragments are digested by methylation-sensitive restriction enzymes. The pattern of the fragment is visible as spots on two-dimensional gel electrophoresis and detected through the radiation of labeling fragments on films [45] (Fig. 1).

Additionally, these spots in gel electrophoresis could be amplified through PCR and sequencing. RLGS could be applied in whole-genome sequencing approaches. It readily represents the valid identification of hyper- or hypo-methylation status in diseases, identifying the methylation imprinted loci, DMRs in cancers, and differential tissue-specific patterns. Although RLGS is a simple and complex-based technique to detect the comprehensive methylation status of the whole genome, its resolution and adversity are less than those of the recently superseded high quantitative and qualitative methods [26, 44].

#### Methylated CpG island amplification (MCA)

Methylated CpG island amplification (MCA) is a simple method of identification of patterns of CpG island methylation, based on digestion, amplification, and microarray technologies to detect aberrant DNA methylation genome wide in a high-throughput manner. The methylated DNA is digested through non-methylation and methylation-sensitive restriction endonucleases such as *HpaII*/*MspI*, respectively*.* Then, the digested fragments are ligated by specific adaptors. The DNA fragments amplified with adaptors by PCR are labeled with fluorochromes. The signals received from a microarray platform are detected from MCA samples but not in the control. The sensitivity of the method is lower than other enzyme-based, MeDIP, and MBD-based techniques (Fig. 3). Additionally, the MCA method is limited to the known CpG regions of the genome and efficiency of the analysis of the genome-wide throughout studies is not sufficient. Recently, this method has been involved in representational difference analysis (RDA) and array hybridization [46] (Fig. 1).

### Bisulfite treatment-based techniques

For the first time, Frommer and colleagues, in 1992, introduced sodium bisulfite deamination as a technique to discriminate between methylated and unmethylated cytosine nucleotides of DNA [47]. Although the bisulfite treatment of DNA leads to deamination of unmethylated cytosine and converts it to uracil, it does not affect the methylated cytosine on CpGs of the genome. Therefore, after bisulfite treatment, the unmodified cytosines are the methylated region of the genome. This pretreatment establishes the basis of many putative techniques in methylation detection and analysis [15]. The techniques involving methylation of specific locus analysis include methylation-specific PCR (MSP), methylation bisulfite sequencing (MBS), pyrosequencing, high-resolution melting (HRM), and real-time PCR. Additionally, the bisulfite-based genome-wide methylation analysis is followed by next-generation sequencing (NGS) and high throughput analysis of bisulfited DNA. The sulfonation of cytosine in the CpGs and non-CpGs induces hydrolytic deamination in C-4 of cytosine and turns into uracil-sulfonate, while methylated cytosines remain unchanged [5]. The methyl group at C5 of 5-methylcytosine is out of reach and remains intact during bisulfite treatment [6]. This step is critical to achieving satisfactory quality and further repeatability [13, 48].

The foundation of the mentioned methods is a chemical modification on cytosine in the single-stranded DNA. Hence the denaturation of DNA during bisulfite treatment is indispensable. Additionally, following treatment and denaturation, the single strand DNA is not complemented. Therefore, one of the DNA strands is useful in bioinformatics analysis evaluations for the performance of downstream techniques such as amplification, sequencing, and array. Generally, the quality (extraction method) and quantity (< 2 µg) of purified DNA and the pH of the reaction are fundamental for application of the subject to bisulfite solution. Incomplete bisulfite treatment is the major problem in bisulfite-related methods; resolving this, Zymo and et al. provided a complete commercial bisulfite research kit*.*

Methods for DNA methylation investigations are generally organized in three main sections: (1) DNA preparation, (2) bisulfite treatment, and (3) identification of methylated regions (PCR, sequencing, or enzymatic digestion). There are several common methodological considerations, such as the scale of the study (high-, medium- or low-throughput), amount of initial DNA, cost and time [49], sensitivity, and accuracy [50], qualitative or quantitative methods [51]. Hence, the common challenges and technical tips for each basic method, such as PCR, sequencing, and enzymatic digestion reactions, should be considered for optimization of unbiased and specific PCR reactions [52], avoiding incomplete or non-specific enzymatic digestions, cost-effectivity, and troubleshooting of cloning and sequencing. Careful consideration is important for the optimization of DNA denaturation status, bisulfite treatment, incubation condition, and assessment of modified DNA after bisulfite treatment [18].

Following DNA preparation, bisulfite treatment is the next step. Cell culture studies, biopsy preparations, and body fluids will require cell separation and DNA extraction and purification. In addition to these studies, some prefer in situ analysis of the cells in intact tissues. These methods will be discussed below in more detail.

#### Methylation-specific PCR (MSP)

**Fig. 2:**
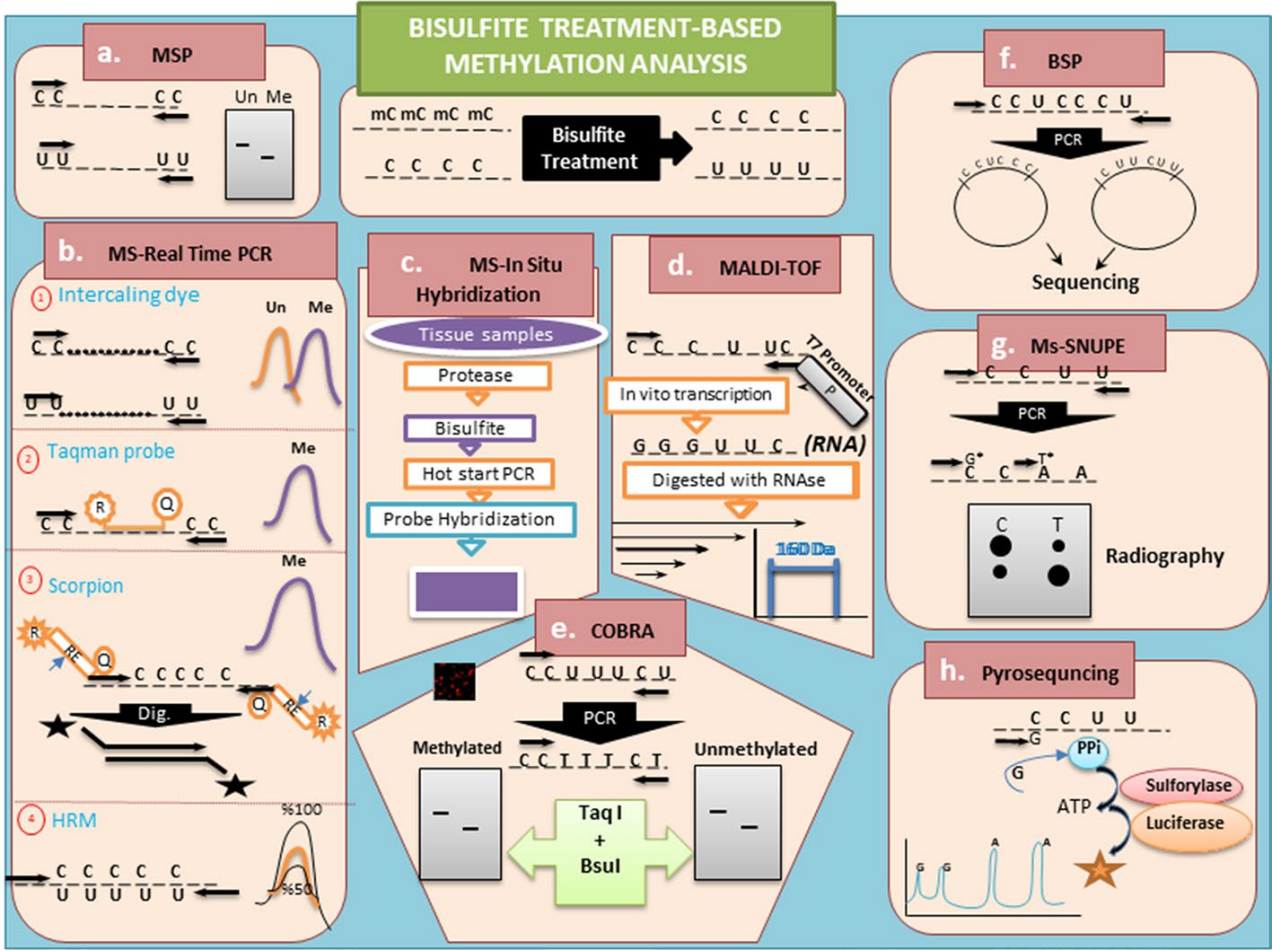
The various methylation analysis techniques used for pre-treatment through bisulfite reaction. **a** MSP (methylation specific PCR), **b** MS-real time PCR (methylation specific-real time PCR), **c** MS-in situ hybridization (methylation-specific PCR in situ hybridization), **d** MALDI-TOF (matrix-assisted laser desorption ionization time-of-flight), **e** COBRA (combined bisulfite restriction analysis), **f** BSP (bisulfite sequencing PCR), **g** Ms-SNuPE (methylation-sensitive single nucleotide primer extension) and h. bisulfited DNA pyrosequencing

Methylation-specific PCR (MSP) is the simplest method for bisulfite modification that is coupled with an amplification step [53]. In this method, two pairs of different primers are utilized for investigating the distinction status of DNA methylation and unmethylation in a certain region. One of the pairs is specified for methylated status (M primer), and the other one belongs to unmethylated status (U primer). Primers should possess at least one or two CpGs at the 3ʹ end for high specificity. Based on several reports, CG-rich regions limit the length of the PCR products; accordingly, the optimum length should be up to 400 bp [16]. Notably, this method evaluates only the methylation primer-localized cytosines. The results are assessed by gel electrophoresis. Additionally, the heterogeneity of methylation is a further matter in amplification that refers to a lack of identical DNA template or methylation heterogeneity in the DNA of cells. As shown in Fig. 2, MSP identified all methylation sites through the target fragment, which is flanked and amplified by primers. Although bisulfite sequencing PCR (BSP) is a preferred method for site-specific methylation assessments, to avoid mixing of templates and heterogenicity, cloning or re-amplification of the region of interest should be performed before sequencing [53, 54].

A further concern, mentioned above, is the existence of two noncomplementary strands of DNA after bisulfite incubation on PCR reaction, one of which would be a template. The primers should not bind to an undesirable strand of DNA in this approach. It seems that the reverse primer is initially attached and amplified, then the forward primer involves amplification, based on 5ʹ–3ʹ orientation. Sometimes primers are attached to an undesirable strand of DNA, which is a drawback of this method.

#### Methylation-specific PCR in situ hybridization (MSP-ISH)

MSP applies to specific tissues and cell communities, because of the variation of the epigenetic patterns, even within a particular cell population. Therefore, in situ analysis of methylation on the intact tissue slices (paraffin or formalin) can be more informative as a biomarker (Fig. 2) [55]. In such cases, it is possible to identify multiple copies of a target sequence at the single-cell level. Therefore, there is no DNA extraction procedure, but there are some protease treatments to expose DNA to bisulfite treatment. Then, in situ MSP is performed, following a conventional PCR amplification step. The first PCR amplifies the target fragment to achieve a high intensity for visualization of methylation-specific PCR probes. Although this method is more challenging than the bisulfite-based methods, it provides more information concerning the quality and quantity of DNA methylation. The size of the probe, concentration, technique of labeling, and optimized hybridization can have an impact on obtaining good results. As well as in situ hybridization, immunohistochemistry (IHC) assays are also important to obtain adequate results [55].

#### Bisulfite sequencing PCR (BSP)

Bisulfite sequencing PCR (BSP) is one of the most common methods in methylation studies, especially for the fragments of genomes [7]. Subsequent bisulfite treatment of DNA, amplification, and sequencing or direct Sanger sequencing are done via primers without CpG sites, because they simultaneously amplify methylated and non-methylated single-strand DNA (ssDNA). However, the heterogeneity of CpGs of the region of interest remains a problem in detection. Overcoming the heterogenicity in the sequencing procedure, the fragments should be homogenized using cloning or reamplification or nested PCR techniques. Following the sequencing of the region of interest, the sequence alignments are performed with the original sequence discerning the methylated CpG [53, 56, 57] (Fig. 2).

#### Methylation sensitive real-time PCR (MS-RT PCR)

The conventional PCR-based methods are not sufficiently quantitative to determine the methylated template in the region of interest [16]. There are some methods based on real-time PCR and bisulfite treatment for the detection of methylation quality and quantity to overcome such limitations (Fig. 2). Considering this issue, researchers developed MS-RT PCR according to TaqMan technology, for example, FAM as a reporter and TAMERA as a quencher or using intercalating dyes such as Cyber green and Cyto 9, etc*.* In the methyLight approaches, there are TaqMan probes to elevate the specificity of the methylation region of interest. The primers flanking the target region utilize subsequent bisulfite treatment, in a real-time reaction. It is important to have a balanced ratio of amplified sequences (methylated and non-methylated) to obtain the right pictures of the methylation patterns. Other achievements concerning the TaqMan probes include methylation-sensitive fluorescent amplicon generation (MS-FLAG) and HeavyMethyl, which promotes the mentioned technology. In the MS-FLAG approach, a pair of specific primers with unique properties is designed for binding to the methylated CpG of the target region. Primers contain a further oligonucleotide on 5ʹ end labeling with a quencher and reporter that are separated by the recognition sequence of the thermostable *PspGI* endonuclease. During amplification, the *PspGI* enzyme cuts double-stranded recognition sites and releases the quencher to generate fluorescence, whereas the unmethylated region is without a PCR product [1, 16]. The next method is HeavyMethyl, related to the presence of oligonucleotide blockers, which bind to the unmethylated CpGs, not to the methylated one, on PCR reaction [58]. Because of the blocker’s presence, the probes are not able to bind to the unmethylated CpGs and are not amplified, whereas probes bind to the methylated CpG and release fluorescent light during amplification [16, 58]. Concerning intercalating dye-based approaches, two technologies have been developed, methylation-sensitive melt curve (MS-MCA) and sensitive melting analysis after real-time methylation-specific PCR (SMART-MSP), which are as the same as methylation-specific PCR with intercalating dyes such as cyber green [58]. The designed primers of MS-MCA are independent of methylated CpGs, while SMART-MSP requires methylation-specific primers. The quality and quantity of the methylation status, the discrimination of methylated and unmethylated strands, and the heterogeneity of methylation are evaluated through melting and amplification curves [16]. Although real-time PCR properties promote the detection of methylation and solved some problems of them, further problems come from the template heterogenicity, with the biased amplification of the unmethylated template [58] (Fig. 2).

#### High-resolution melting (HRM)

High-resolution melting (HRM) is the other quantitative method based on bisulfite and real-time PCR features derived from the melting curve, which related to Tm of the nucleotide context on the product. This method, for the first time, introduced detection of the SNPs of DNA. The methylation status of amplicons is directly determined via the descending temperature during denaturation to renaturation, liberation, and use of intercalating dye. Any modified methylated and unmethylated CpGs, by bisulfite conversion affects the melting curve of the product. In this method, there are commercial methylated and unmethylated DNA for controlling the methylation status of the region of interest, and the methylation curves of samples are evaluated in comparison with DNA controls. Although the method approach highlights the methylation percent of region of interest, it does not give any information concerning methylated CpGs in this region. As mentioned, the biasing of amplification in methylated and unmethylated DNA as a template are a further problem that minor changes in primer design can optimize [59–61] (Fig. 2).

#### Bisulfited DNA pyrosequencing

M. Ronaghi and colleagues were the pioneer designers of pyrosequencing [62], then developed it to analyze bisulfite-treated DNA. In this method, the sequence of bisulfite-converted fragments (specific CpG sites) is identified using pyrosequencing. The luminometric detection is obtained from pyrophosphate release that is shown using each nucleotide. The conversion of C-to-T bases, in bisulfited DNA at the region of interest, can be quantitatively assayed, regarding incorporated C or T in amplification. In pyrosequencing, various DNA copies are amplified in a single reaction that facilitates the assessment of methylation. Additionally, it is suitable for high-throughput screening analysis in methylation, whereas the analysis of a short length is a disadvantage. Recently, Wong et al. improved the mentioned route through using allele-specific primers incorporating single-nucleotide polymorphisms to separate analysis of maternal and paternal alleles. Although this method is efficient, reliable, and flexible, the technology cost is not reasonable [63, 64] (Fig. 2).

#### Methylation-sensitive single nucleotide primer extension (SNuPE)

The method of SNuPE is a fast quantitative method in genetic engineering [8] that has been applied for site-specific methylation analysis. After DNA treatment with bisulfite, the target region will be amplified via free CpG site primers. Using this method, we can only study the methylation status of a CpG site with two oligonucleotides. Instead of total dNTPs, the labeled dTTP and dCTP are added to each reaction separately. This technique is called the methylation-sensitive single nucleotide primer extension method (Ms-SNuPE) (Fig. 1) [9]. Therefore, multiplexing is possible by considering a different sequence and length for each oligo. PCR amplification of the target region is performed. To obtain a sufficient quantity of modified PCR product, it should be purified from dNTPs and concentrated. The oligonucleotides for the second amplification are related to further specific primers such as MSP. The mentioned amplification step is only the addition of one base pair to primers via existing dTTP or dCTP nucleotides. Acrylamide gel electrophoresis can help to analyze the result. Since the method does not need sequencing or cloning procedures, it is very quick to check specifically targeted methylations. Ion-pair reverse phase HPLC is used for the detection of extended oligonucleotides and is named the SIRPH method (SNuPE with IP-RP-HPLC) [10]. Some have used MALDI-TOF spectrometry to investigate the differences between extended nucleotides [11] (Fig. 2).

#### Matrix-assisted laser desorption ionization time-of-flight (MALDI-TOF)

Recently Ehrich et al. reported a technique based on bisulfite conversion for the analysis of methylation called MALDI-TOF–MS. In this method, bisulfite-treated DNA is amplified by primers for which the reverse primers include a promoter-tagged T7. Then, in vitro transcription of the PCR product is performed and conversion of the RNA strand in the region of interest. Next, transcribed and amplified RNAs are digested by *RNase A*. *RNase A* cuts RNAs, specifically from the cytosine and uracil residues. The usage of dCTP during cleavage leads to digestion specifically in uracil nucleotides by *RNase A*. The cleaved fragments of RNA can be analyzed via MALDI-TOF. The mass of converted C-to-T through bisulfite treatment is recognized in mass spectrometry as a mass difference of 16 Da. The Epityper assay is valid, fast, quantitative, and reproducible to analyze the regions of interest to cover the mixed population of methylated DNA and methylation heterogeneity in the region of interest [21, 65, 66]. This method is suitable for analyzing the methylation status-related CpG islands to evaluate biomarkers of many gene loci or DMRs on the genome, whereas large-scale genome-wide methylation analysis is not worthwhile for this method, and is very expensive. Additionally, the discrimination of 5-mC from 5-hmC is not done with the mentioned method [57, 65, 67] (Fig. 2).

#### Combined bisulfite restriction analysis (COBRA)

**Fig. 3:**
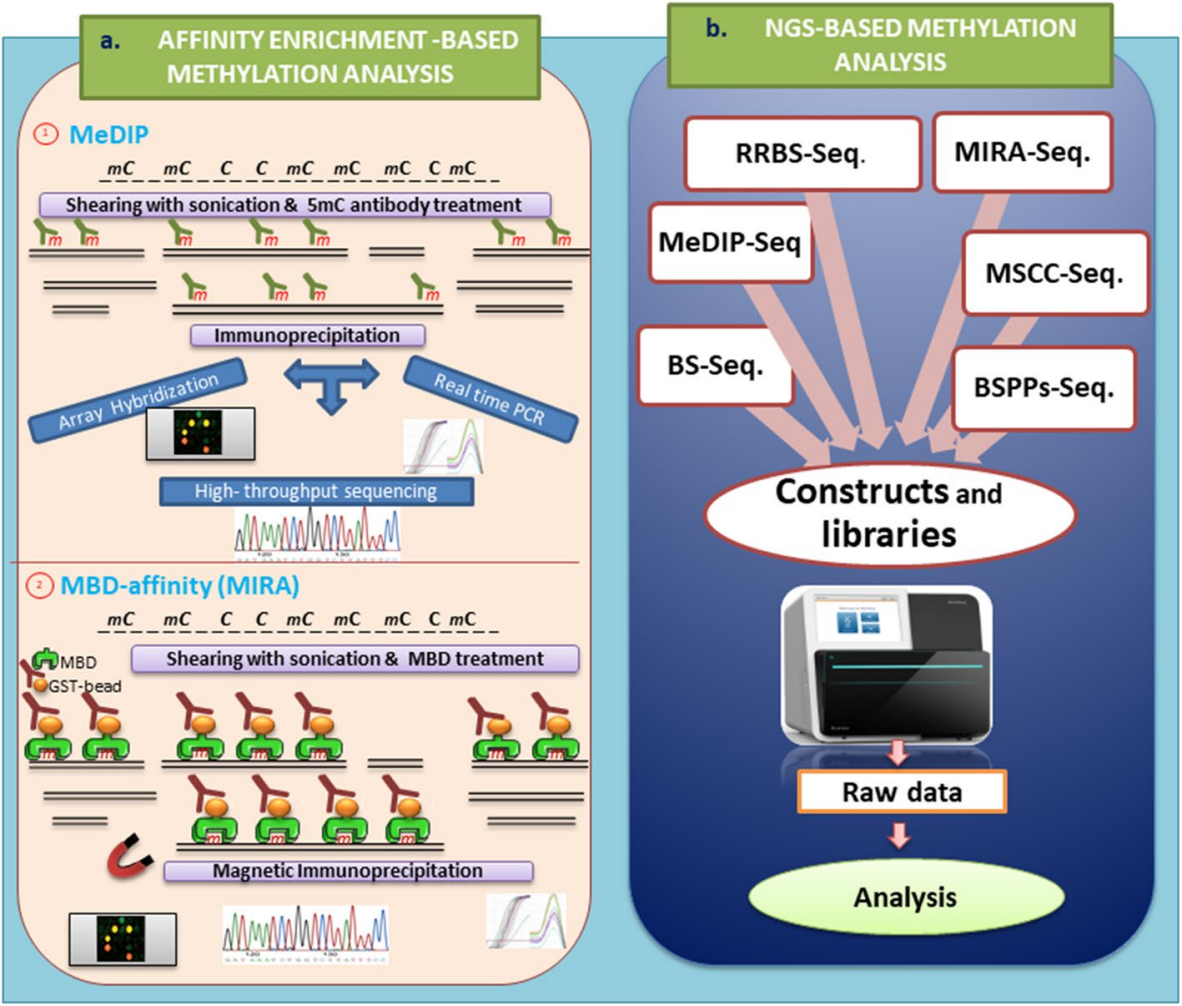
**a** Methylation affinity enrichment is a further method to discriminate methylation status including 1. MeDIP (methylated DNA immunoprecipitation) and 2. MBD-affinity (methyl binding domain-affinity). **b** Also, next generation sequencing of methylation approaches include RRBS-Seq., MIRA-Seq., MeDIP-Seq., MSCC-Seq., BS-Seq., BSPPs-Seq

Combined bisulfite restriction analysis (COBRA) is a method for the detection of locus-specific methylation in which both bisulfite treatment and restriction endonuclease are applied. In this application, it consists of three steps, including DNA treatment with bisulfite, PCR amplification, and restriction endonuclease digestion. In this procedure, the cytosine converts to uracil, and during the amplification on PCR, uracil turns to thymine. Therefore, it can destroy or reestablish the recognition site of the endonuclease, whereas the methylated cytosines of CpGs belonging to the recognition site of an endonuclease remain unchanged through bisulfite treatment and PCR amplification. The bisulfite conversion leads to the generation of the heterogeneous strands of DNA fragments containing cytosines and thymines at specific positions, in which the cytosines had previously been methylated and unmethylated, respectively. The PCR products are cleaved by some related restriction endonuclease, such as *BsiWI*, *MluI*, and *TaqI*. Gel electrophoresis is performed to detect the PCR product fragments. The quantification of methylation status can be evaluated through comparing the ratio between the digested and remaining PCR products for determining the rate of methylated and unmethylated cytosines in the region of interest in each strand of DNA, separately. Although the mentioned technique is reliable for the quantity and quality of the methylation locus, the restriction enzyme problems for analysis are a limitation of this assay [68, 69] (Figs. 2, 3).

#### Disadvantages of bisulfite treatment

In bisulfite reaction, both sense and non-sense strands of DNA are treated through this reaction that leads to establishing non-complementary DNA. Therefore, one of them applies to downstream approaches for detection, whereas another strand may be a source of contamination in associated methods. Hence, it is important to select the proper strand for methylation studies. Next, the extracted DNA derived from various cell populations and treated with bisulfite reaction make the detection of methylation status problematic. Sometimes the bisulfite treatment of DNA is performed incompletely, which affects the downstream related approaches. Furthermore, the amplification rate of methylated and unmethylated strands is different and accompanied by a bias that could disrupt the quantity of methylation status [56, 57].

Despite downstream bisulfite-based method problems, the quantity and quality of inputted DNA and quality of bisulfite reaction are vital in this process. It can be noted that the incomplete denaturation before bisulfite reaction, incubation times, PH calibration (= 5.4), sensitivity to degradation of DNA, and the quantity of purified DNA in bisulfite treatment are problematic in this regard. Additionally, bisulfite pretreatment of DNA cannot discriminate 5mC and 5hmC on CpG dinucleotides of DNA. Subsequently, 5hmC is converted to cytosine-5-methyl sulfonate that it is read as a cytosine in sequencing and amplification. Therefore, the bisulfite-pretreatment related methods are not more reliable for a 5mC pattern of the genome [47, 57].

### Affinity-based technology (methyl binding domain- and antibody precipitation)

Various studies have shown that a methyl group could be targeted via some proteins collaborating with transcription factors, such as methyl binding domain (MBD) portions within cells. On the other hand, it was also demonstrated that some antibodies could act as an affinity enrichment to the methyl group. Therefore, both ways can apply as a pretreatment to discriminate methyl-cytosine from another one within the genome. It should be noted that methylated CpG-rich regions bind to proteins more than methylated CpG-poor sequences. The affinity enrichment approach isolates the methylated fragment from the unmethylated one. It is usually performed through antibody immunoprecipitation of MBD proteins [70, 71].

Indeed, the two mentioned approaches, methylated DNA immunoprecipitation (MeDIP) and MBD, are discussed in this part, because of the affinity enrichment and immunoprecipitation of methylated regions of both genomes (Fig. 3). MeDIP targets the single strand molecules, whereas MBD could capture the double-stranded methylated DNA. These methods can determine both genome-wide methylations of DMRs throughout the genome [18, 27]. Furthermore, the MeDIP and MBD affinity approaches are non-sensitive to the impurity of initial inputted DNA related to the others [72]. Although these approaches are efficient and rapid tools for the following techniques, such as PCR, array-hybridization, and next-generation sequencing in a complex genome, they are unable to discriminate methylation status of CpG islands or a single CpG dinucleotide [24, 70]. The difference between MeDIP and MBD techniques is that the specific antibody in MeDIP binds to the single-stranded DNA after denaturation and is useful for the detection of low CpG density, whereas the MBD-based approach captures double-stranded methylated fragments with higher CpG density, especially CpG islands [73].

### Methylated DNA immunoprecipitation (MeDIP)

MeDIP is a multipurpose approach with antibodies against methyl groups that analyses all downstream methylation from low- to large-scale [24, 74]. This method was first reported by Weber M. et al. in 2005, stating that it was only performed with the aim of helping to evaluate genome-wide methylation [27]. Although the mentioned methods are successful in the determination of typing and profiling methylation status, the methylome pattern or DMR status is variable from cell types to tissues [24].

In this approach, the extracted DNA, in a small amount (~ 2 μg), is sheared by sonication because it is fast and without restriction enzyme digestion biases to obtain random length fragments between 300 and 1000 base pairs. The fragments with shorter lengths affect the promoting efficiency of this method and its precipitation step. Additionally, the binding monoclonal 5mC antibodies are impacted not only by prepared fragment size but also complete denaturation and production of single-stranded DNAs of these fragments. After incubation of fragments with specific antibodies, such immunoprecipitation routes are used. For example, the magnetic beads bind to antibodies related to 5mC, and unmethylated DNA is isolated from them. Then, antibody-binding methylated DNAs are treated via proteinase *K* to remove the antibodies and leave the methylated DNA fragments. These DNAs could apply to downstream techniques, such as from traditional PCR to next-generation sequence approaches in the detection of methylation; the most common one is related to the combination with DNA microarrays for high-throughput DNA methylation profiling. Additionally, it is the putative approach to apply to methylation, regardless of the specific sequence or non-CpG methyl-cytosine in the genome of other organisms, such as mouse, chimp, *Arabidopsis thaliana*, and *Neurospora crassa*, because the methyl-cytosine context is different in the various organisms. As known, this method can discriminate 5mC from 5hmC on the genome, because of specificity in applied antibody for 5mC [24, 70].

The resolution and sensitivity of the MeDIP technique are associated with coupling downstream applicable approaches, such as high-resolution array hybridization, high-throughput sequencing MeDIP-seq, MeDIP-ChIP, and quantitative real-time PCR (qPCR) as well as traditional PCR, which involve different data analyses. For example, qMeDIP or real-time quantitative PCR, and each one of the related techniques, are relatively quick and cost-effective for the detection of specific loci and target genes, with specific primers of interest [16]. The normalization of qMeDIP is performed through the imprinted gene *H19* and the housekeeping gene *GAPDH* as positive and negative controls, respectively.

### MBD protein affinity approach

MBD proteins translate the methylation status of DNA to chromatin-related complexes, involving transcription. Therefore, it was shown that the mentioned proteins potentially could be applied for the detection of DNA methylation [75]. In this case, MBD2b is more powerful than MBD enrichment, because the heterodimer of MBD2b has a higher affinity with methyl-CpG in double-stranded DNA and independent sequences. It has been proven that the MBD3L1 proteins could enhance the binding affinity. This method, because of binding to at least methylated CpG sites, not only is suitable for finding DMRs in the genome but also is applicable for genome-wide methylation studies [73]. The purified DNA is cut to generate a shorter fragment. The methylated fragment DNA, after capture by MBD proteins, is isolated through precipitation methods and pulldown. The optimized approach, according to this technique, is methylated-CpG island recovery assay (MIRA) that applies simultaneously MBD2b and MBD3L1 for establishing specificity and enhancement, respectively. The extracted DNA is digested by *MseI* endonuclease and treated with the MBD2b/MBD3L1 complex. The captured DNA fragments are pulled down via MBD2b GST tagging and glutathione beads. Additionally, mC immunoprecipitation (mCIP) is another MBD-based approach in which the recombinant MBD2 interacts with the Fc tail of antibodies to precipitate methylated DNA. The linker ligation and amplification of methylated fragments could add the affinity enrichment methods with array hybridization to enhance resolution and efficiency of detection including the MethylCap kit (Diagenode), and MethylCollector Ultra kit (Active Motif) with distinct fluorescent dye labeling of inputted and enriched fragments [24, 70].

There are some parameters concerning the quality and quantity of input DNAs in all of the enzyme-, bisulfite- and affinity-based methods. According to various studies, the DNA requirement for restriction enzyme is more than 2 µg, whereas bisulfite pretreatment requires a lower amount. The efficacy of the mentioned pretreatments depends on the purity and source of extracted DNA samples [13, 47], for example, fresh, paraffin-embedded, and formalin-fixed DNA samples. Some microarray approaches were developed and overcame this problem.

### Toward next-generation sequencing and methylation analysis

Despite development of methylation analysis approaches, the aims of valid methylation studies, concerning mapping and profiling genome-wide levels, are different because of natural heterogeneity throughout the genome [47]. The comprehensive typing and profiling approaches, associated with DNA methylation, were discussed in the previous section. Some pretreatment analyses for assessing genome-wide methylation, such as RLGS, DMH, methylated CpG island amplification and microarray (MCAM), reduced representation bisulfite sequencing (RRBS), and whole-genome bisulfite sequencing (WGBS), were related to MRE and bisulfite pretreatment [24]. Affinity enrichment techniques through MBD and MeDIP capture are useful genome-wide methylation studies. According to developed methods, the upstream NGS-based approaches again refer to formerly mentioned approaches in which there is various pretreatment of DNA for determining methylation. In this regard, some techniques have recently been developed for the mapping of DNA methylation on a genome-wide scale. Some of them are based on traditional restriction enzymes such as methylation-sensitive (e.g., *HpaII* and *NotI*) or methylation-specific (e.g., *McrBC*) enzymes, whereas the minority (< 5%) of CpG regions are available to these enzymes for recognition. The best methods for targeting more than 90% of methylated CpGs are MBD and MeDIP approaches because the recognition sites are more specific. Therefore, the methylation-sensitive endonucleases are secondary in application to genome-wide scale analysis = Reduced representation bisulfite sequencing (RRBS) and Methyl-Seq/HELP-seq are NGS-related approaches and very critical for a genome-wide study that belongs to MRE based-methods. Although the recognition sites of MRE are restricted in comparison with sensitivity and specificity of MeDIP and MBD approaches, the quality and coverage of DNA of current restriction enzyme-based techniques are better suited for read-out by next-generation sequencing techniques in comparison with others [14, 27].

Comparison with deep sequencing approaches with array-based platforms shows that there are some disadvantages concerning array-based approaches, including lower resolution and having a problem to distinguish the methylation of the repetitive element on the genome fragments. To the best of our knowledge, the resolution and coverage of deep sequencing in comparison with the traditional enzyme-, bisulfite—and affinity-based methods overcome several limitations. Applying the mentioned approaches based on NGS showed efficient implementation and attenuated interpretation of the methylation sequencing basis [70].

One of the traditional methylation analyses is bisulfite sequencing in the region of interest with precise and nucleotide base-level resolution. This method can be performed at a genome-wide level, namely WGBS. In this procedure, the extracted DNA is digested into several smaller fragments. An adenine nucleotide is surcharged to the 3ʹ ends as poly ‘A’ tailing. Then, methylated sequencing adaptors are ligated to fragments. After bisulfite conversion of DNA fragments, the selection of fragment size is performed and they are purified through gel electrophoresis. The selected fragments are amplified through PCR to prepare a library for sequencing [16].

As mentioned, RRBS is an accurate and cost-effective approach for genome-wide methylation mapping at a base resolution level. In RRBS, *MspI* or *Bgl II* restriction enzymes digest the genomic DNA and cut it into nearly 200 bp fragments as a library. The fragments are end-repaired on their 3ʹ-tails through ‘A’ nucleotides, which enables them to ligate to the adaptors. This method is appropriate for quality, quantity, and sample source assessments [16, 18].

MeDIP-seq, as a next-generation approach, involves short-read sequencing through pyrosequencing or Illumina. After digestion through sonication, DNA fragments are denatured. After the methylated fragments are obtained from 5mC-antibody precipitation, high-throughput sequencing of a large number of short reads is performed. After sequence alignment with the reference genome, the sequence reading defines the mapping of fragments. The extended reads are applied to evaluate the methylation status. The analysis applying tools such as DAVID and GoSeq has some bias concerning high-throughput methylation. The data of methylation obtained by PCR are analyzed through a statistical model using a control. This method covers more than 70% of CpGs of the genome [15].

High-throughput DNA methylation analysis approaches mostly belong to high-resolution DNA microarrays (MeDIP-chip) and next-generation sequencing (MeDIP-seq). There are some problems concerning data generation of MeDIP, especially analyzing methylation density on the CpG-poor fragment of regions that refer to immunoprecipitation efficiency. In this regard, Down et al. developed a Bayesian tool for methylation analysis (Batman) to determine absolute methylation status from data obtained from MeDIP results from methylated CpG regions that cover overall CpGs with higher efficiency in the fragments of the genome. Altogether, MeDIP and another affinity-based methods permit efficient and rapid determination in methylation analysis throughout the genome [70, 73].

A further approach with more success in high-throughput sequencing belongs to MBD enrichment based methods and is called methylated-CGI recovery assay (MIRA). In this procedure, the following purified DNA fragments are performed by MBD2b and MBD3L1 proteins after sonication. Treatment of DNA via glutathione-S-transferase (*GST*)-tagged MBD2b and histidine (HIS)-tagged MBD3L1 proteins provides the binding of methylated double-stranded of fragments. Then, magnetic glutathione-coated particles precipitate enriched methylated DNA without denaturation. Finally, the library preparation is done for sequencing. MIRA is a cost-efficient method for high throughput NGS technologies to discriminate human DMRs on a genome-wide scale [18].

Additionally, there exists a Methyl-Seq method via hybridization enrichment in which fragments with CpG are converted through bisulfite. After the fragmentation, DNA ligates into the adaptors. The bisulfited fragments hybridize to RNA baits, designed for targeting more than 3.5 million CpG, such as Roche company kits (SeqCap Epi CpGiant Enrichment Kit). Although the bisulfited DNA converts before hybridization in the RRBS method, it seems that Roche’s enrichment strategy in the mentioned manner attenuated biased. Additionally, the bisulfite treatment is more biased than the enrichment method, where both of them experimentally have the same ability to detect DMRs of the genome. Further studies showed that both enrichment and bisulfite conversion, such as RRBS and Illumina’s 450 K array, respectively, obtain the same efficiency of DMR determination results [18].

Roche’s enrichment approach is efficient and more advantageous because a single nucleotide on DNA differentiates and applies to the methylation status of DMRs. In this method, the methylated and unmethylated fractions are isolated via digestion with methylation-sensitive restriction enzymes. The short fragments are considered as unmethylated fragments. Then, the large methylated fragment is isolated and amplified through the specific *phi29* polymerase. Subsequently, they are treated with the same restriction enzyme once again to get methylated fragments. Suppression of the amplification of unmethylated fragments is performed through applying blocking adaptors [52].

The high-throughput methylation study has a problem in data analysis. Regarding the accuracy in alignment of repetitive regions and short readers of the genome, bisulfite sequencing requires a significant adaption to higher resolution. Some tools for gene-set analysis, such as GoSeq and DAVID, improve bias in high-throughput methylation data by applying statistical models [56].

The downstream approaches in NGS platforms are different, with some advantages. Ultra-deep sequencing of Roche 454 platform performs real-time pyrosequencing in more than 100 products in a single run with more than 1600 reads per locus coverage [24].

After tissue fixation, protease treatment prepares DNA for the bisulfite treatment. Afterward, two sequential amplification steps are performed. The first amplification reaction is a normal conventional PCR, but the second reaction is a methylation-sensitive reaction via labeled probes.

### 5-Hydroxymethylcytosine (5hmC) detection

As mentioned, the demethylation of DNA is performed by converting 5mC to hydroxy-methyl-cytosine (5hmC) through TET family enzymes. 5hmC is a new epigenetic marker that recently demonstrated most roles in various mechanisms of cells. Therefore, it seems the detection of 5hmC profile could be important in some diseases [16, 23, 76].

There are some types of approaches for 5hmC analysis of the interested region and throughout the genome. One of them is a relation to glucosyltransferase and endonuclease enzymes in which glucosyltransferase firstly modifies 5hmC as the only substrate on the genome. Then, it is followed by *MspI* digestion. The glycosylated 5hmC is resistant to cleavage on the recognition site of the mentioned enzyme so it could be applied in both NGS and qPCR assay [48, 77].

On the other hand, the bisulfite pretreatment is not able to distinguish between 5mC and 5hmC on DNA. This method converts 5hmc to C-5-methyl sulfonate, which counts as a cytosine in sequencing. Therefore, bisulfite-related methods are not reliable for the 5mC pattern of the genome. Additionally, a part of detected 5mCs in the genome is related to 5hmC, which is a false positive for methylation. Thus, there is a recommendation utilizing approaches other than this one. Alternatively, Chuan He et al. recently have promoted Tet-assisted bisulfite sequencing (TAB-seq) that can distinguish between 5mC and 5hmC [78]. Before treatment reaction, the purified DNA serves as a substrate to TET enzymes, converts 5hmCs to 5fmC, and finally U. Therefore, they are read as ‘T’ on sequencing. In this regard, a further approach, called oxidative bisulfite sequencing (oxBS seq), could be applied to discriminate 5hmC from 5mC, and 5hmC level can also be quantifiable by chemical oxidation and compared with BS sequencing [79]. Additionally, sometimes it is essential to evaluate hypomethylation regions on the genome, with some methods, such as a Hypo-Methyl collector kit, which should be developed. Unmethylated CpG islands target the CXXC binding domain, derived from mouse Mbd1 protein [16, 48, 77].

## Overview of histone modification analysis

In eukaryotes, covalent modifications on the core histones play a basic role in chromatin regulation and gene expression. Histone modifications have essential regulatory roles in different types of cell functions such as DNA replication, chromosomal separation, repair, and epigenetic gene silencing [80, 81]. For example, methylation on each lysine (K) of histone 3 (H3) can lead to different and even inverse outcomes [7, 82, 83]. Probably, methylation on K4 of histone H3 inhibits binding of the chromatin remodeling suppressor complex to chromatin. Conversely, methylation in K4 and K27 is involved in suppression of gene expression among chromosomal domains. Activator/suppressor heritable states with these two methylated lysines can lead to detection by particular nuclear proteins [13]. Also, histone acetylation regulates heterochromatin formation. Acetylation of H4-K16 operates as a barrier for heterochromatin components’ extension [84]. Many studies have revealed some useful information for decoding functions of specific histone modification including paracentric chromosomal condensation [85, 86], and X-inactivation (X-chromosome inactivation) [7, 87, 88] in mammals, which is methylated H3-K9 and H3, H4 acetylation. However, there is less information about how specific histone modifications are organized around particular genes or chromosomal regions in mammalian model systems.

Besides packaging, chromatin structure modulates gene expression, mainly through post-translational modifications of histones. These modifications are site-specific and dramatically change many biological processes [13]. The post-translational modifications of histones convey a unique characteristic to the nucleosome that modulates the binding and activities of other proteins that interact with DNA and the histones. In the following sections, we will review the most common histone molecule assessment methods based on” (1) chromatin immunoprecipitation assay, (2) modified ChIP-based methods, (3) site-specific analysis of histone modifications.

### Chromatin immunoprecipitation assay

The current understanding of the epigenetic function of chromatin is highly influenced by chromatin immunoprecipitation assay (ChIP assay), which has created a new epigenetic perspective in gene expression, differentiation, and understanding of diseases.

ChIP assay is a prominent technique in mapping the histone modification sites on DNA and the analysis of protein-DNA interactions and transcriptional regulations. Site-specific protein-DNA interactions can be detected using the relative density of the interactions [13, 81]. ChIP is the most common technique for the analysis of histone modifications based on the presence of antibodies for detecting the site-specific histone modifications. It also detects new TF or chromatin-binding proteins and the mapping of histone modifications before/after transcription.

At first, scientists studied ChIP assay for the relation and role of hyperacetylated histones and particular DNA sequences. This method determines the characteristic DNA sequence responsive to specific TFs, and consequently widely identifies protein binding sites in in-vivo and in-vitro experiments [13]. Now, the effective binding of TFs and protein complexes in chromatin remodeling and DNA mapping are broadly analyzed by ChIP assay [89]. ChIP assay usually has three steps: (1) chromatin preparation, (2) immunoprecipitation, and (3) analysis of DNA sequence [13].

#### Chromatin extraction, fragmentation, and fixation

**Fig. 4:**
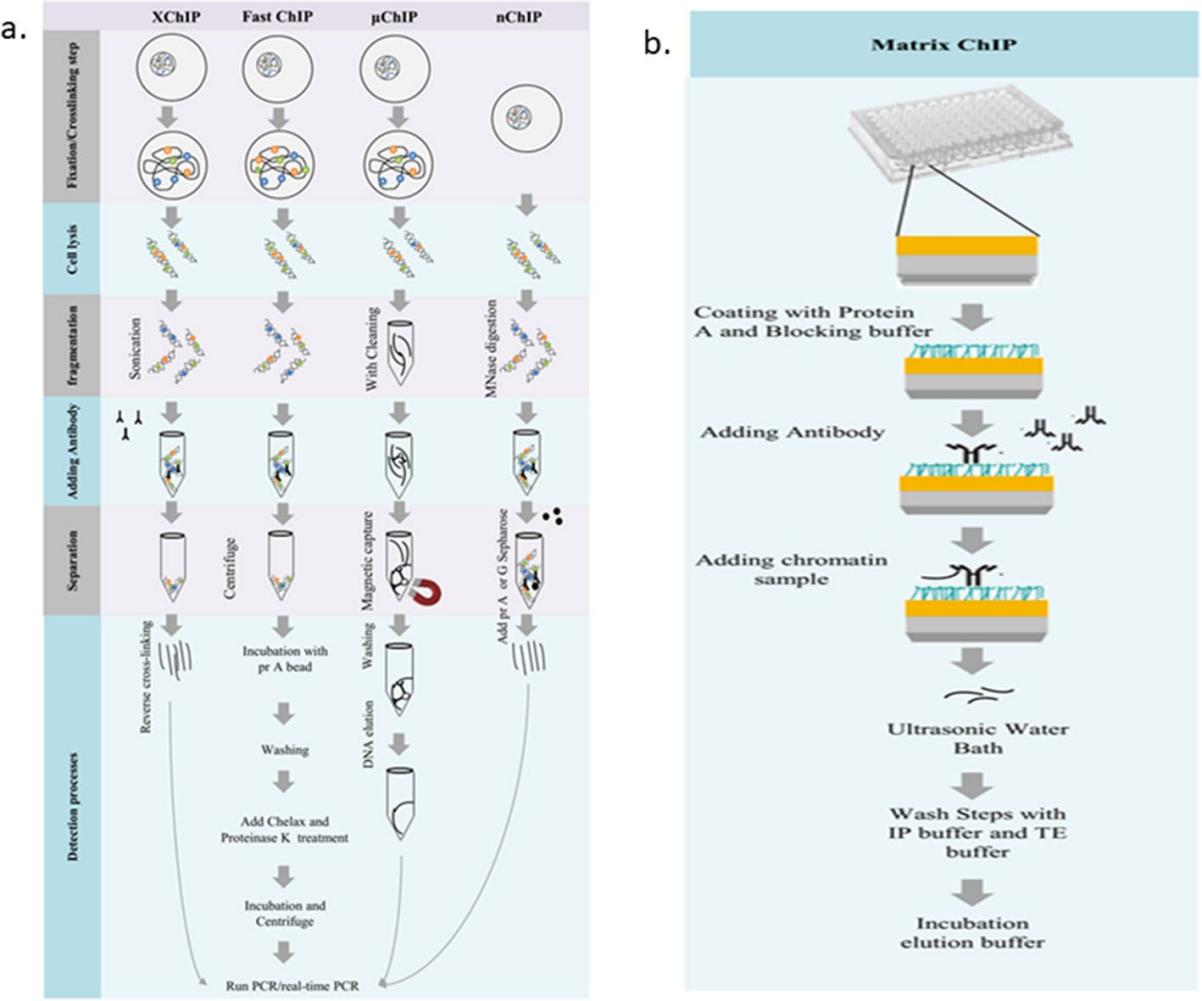
**a** Outline of chromatin immunoprecipitation (ChIP) assays, site-specific analysis or native ChIP (NChIP) and other improved ChIP assays (fast and μ or macro ChIP. In summary, the fixation/crosslinking step is an essential step in the ChIP protocol which is removed by the enzymatic method. After nuclei purification from tissues or cultured cells, chromatin is fractionated with enzymatic or no enzymatic treatments. Then, purified nucleosomes incubated with antiserum are directed against the histone modification of interest. Subsequently, detection is performed by PCR/quantitative PCR (QPCR) technologies. **b** Outline of matrix chromatin immunoprecipitation (Matrix ChIP) assays; Matrix ChIP is an improved version of the classic ChIP assay. Surface-immobilized antibodies in 96-well plates are used. The assay has enhanced antibody binding capacity and minimal sampling for detection of low- and high-affinity proteins in only 1 day

The study of low- and high-affinity protein-DNA interactions needs two different experiments. High-affinity interactions, such as histones, need no treatments after fragmentations. Therefore, native DNA is cleaved by micrococcal nuclease enzyme (*MNase*), which is an enzyme that preferentially cleaves the DNA linkers between the nucleosomes (Fig. 4a). Purified DNA should be fragmented into small pieces using enzymatic, photochemical (UV radiation), and physical (sonication) treatments. This method exploits the native chips (NChIP), which keep the chromatin intact and do not use endogenous nucleases for chromatin degradation, and all steps of the nuclei purification should be done on ice, or at 4 °C.

The advantage of using native epitopes is that the antibody remains intact during the chromatin preparation. Also, native chromatin tends to give high levels of precipitation for a specific histone modification [90]. This method has a slight drawback, as normally it precipitates a small fraction of chromatin and relies on random digestion; it also does not regularly produce small chromatin fragments on the region of interest. In the latter method, sonication breaks chromatins. As previously mentioned, this ChIP analyzes low-affinity interactions. Hence, there is a fixation/crosslinking step carried out by formaldehyde treatment or UV. The nuclei isolation after crosslinking increases the incubation time, and the nuclei isolation before fixation keeps cytoplasmic proteins from interfering. Sometimes low-affinity interacting proteins are analyzed, such as non-histone proteins transcription factors (TFs). Formaldehyde is one of the most common crosslinking reagents that fix low-affinity protein–protein, protein-RNA, and protein-DNA interactions through amino and imino groups of lysine, arginine, and histidine amino acids, reversibly. Crosslinking, which is an essential step in the ChIP protocol, depends on the cell type or tissue and the factor of interest. Also, it is important to use appropriate concentrations and duration of formaldehyde for it. Over-crosslinking can increase immunoprecipitation of contaminant and unrelated fragments. Also, excess formaldehyde may over-crosslink with the epitope surface and interface with proper fragmentation. On the other hand, insufficient crosslinking can cause incomplete fixation and improper DNA fragmentation to less than 500 bp [13, 90–95]. Therefore, the sonicator should be calibrated to achieve the final desired average length of DNA. This method is known cross-linked ChIP (XChIP).

#### Immunoprecipitation of fragmented chromatin

After chromatin preparation (cross-linked or native), specific antibodies are used to target the modification of interest. A suitable amount of antibody can be determined by preliminary immunoprecipitation experiments. There would be special considerations about immunoprecipitation optimization; the excessive amount of antibody causes over-precipitation of DNA. It is necessary to avoid non-specific bindings by control. Additionally, the efficiency of modified histone precipitation depends on the amount of applied antiserum, and the affinity of the antibody to its epitope. Additionally, it also depends on whether the modification is on a common locus of interest in the genome or a rare one. The best precipitation results are usually achieved from rare modifications. The determination of sensitivity and efficiency of PCR is important before immunoprecipitation [13].

#### Analysis of DNA sequences

Immunoprecipitated chromatin fragments are quantified by techniques such as low-throughput experimentations, e.g., polymerase chain reaction (PCR), quantitative PCR (qPCR), and targeted sequencing, as well as high-throughput experiments, such as genome-wide hybridization, DNA microarray (ChIP-on-ChIP), and ChIP sequencing. ChIP is also applied for the mapping of post-transcriptional histone modifications [71].

QPCR is applied for determination of the precise amount of DNA target and increases multiplication via real-time PCR amplification. This method quantifies the initial amount of DNA target, and downstream manipulation is not required. The combination of qPCR with ChIP assay can precisely determine the level of protein binding to a specific region. This method is very useful for the quantification of acetylated H3 and H4 in different regions of a specific locus [96]. It can determine the histone acetylation on distinct nucleosomal DNA [97]. ChIP-qPCR is also beneficial for investigation of the effect of methylation on gene expression and chromatin structure. Acetylated histones are involved with unmethylated DNA regions (not with methylated regions) [13]. Although this method could not map a particular gene site or region of interest in detail, it could analyze the relative amount of protein binding in various samples. The sensitivity of target DNA identification is higher in this assay than the standard ChIP. Furthermore, this method needs a lower cell number (about 3 × 10^6^) in comparison to other ChIP strategies (about 10^8^) [13].

In addition to real-time PCR, duplex-PCR can be carried out to quantify the amount of precipitated chromatin in the site-specific analysis of histone modifications. Duplex-PCR coamplifies the region of interest and a control fragment (e.g., from the actin gene), which are more or less equally amplified.

### Modified-ChIP-based methods

The basic ChIP protocol, as a low-throughput analysis, is significant for study of early embryonal development and cancer biopsies [71, 98, 99]. Despite all the advantages of the ChIP assay, there are some main disadvantages (Table 1). The major limitation is that the ChIP assay needs large amounts of cells (about 10^6^–10^7^ cells) as the initial substrate, which are not easy to obtain from small tissue biopsies, limited stem cells, or embryonic cells. The traditional protocol also is time-consuming and not applicable for parallel analysis. Therefore, several modifications are required to improve the assay, including Carrier ChIP (CChIP), quick and quantitative ChIP (Q^2^ ChIP), MicroChIP (µChIP), Fast ChIP, and Matrix ChIP (Fig. 4a) [81]. Currently, some improvements have made it appropriate for automation.

**Table 1:** General information of different ChIP assays including their advantages and disadvantages are summarized

Technique	Scale	Cells	Advantages	Limitations	References
nChIP (1988)	High throughput	10^6^ ~ 10^7^	Recommended for high-affinity DNA binding proteins Proteins remain intact Good chromatin and protein recovery efficiency Bead-based immunoprecipitation	Time consuming (several days) Labor intensive (several precipitation steps) High variability in results Not recommended for non-histone proteins	[89, 90]
XchIP (1984)	High throughput	10^6^ ~ 10^7^	Recommended for any weakly chromatin-associated proteins such as TFs Recommended where native protein is hard to prepare	Time consuming (several days)	[90]
CChIP	Low throughput	100	Requires as few as 100 cells It takes two to three days	It takes two to three days Highly specific primers are required	[91]
Q2 CHIP	Low throughput	100,000	Reduced steps Enhanced signal to noise ratio	Time consuming (several days)	[92]
MicroChIP/μChIP (2007)	Low throughput	10,000	Applicable for genome-wide studies	Time consuming (several days)	[93]
Fast CHIP (2006)	High throughput	10^6^ ~ 10^7^	The incubation time is decreased Several steps are reduced in protocol Preparation of PCR-ready template is reduced to 1 h	Suitable if only for large cell samples are available	[94]
Matrix ChIP (2008)	High throughput		Enhanced antibody binding capacity Requires minimal sampling Very Fast (1 day) Can detect both Low and High-affinity proteins Automation is possible Includes high sensitivity High reproducibility		[95]

Carrier-ChIP (CChIP) assay immunoprecipitation requires as few as 100 cells, and it lasts for 2–3 days. Carrier chromatins (e.g., from *Drosophila*) are capable of facilitating the method (Table 1). [100]. In this method, the target cell chromatin can be distinct from the external background carrier chromatins, using very specific primers.

As an alternative, quick and quantitative ChIP (Q^2^ ChIP) assay is a method that is appropriate for almost 100,000 cells. A single template genomic DNA can be stored in several dilutions and aliquots in the Q^2^ ChIP method. Reduction of the number of steps, increasing antibody-to-target epitope specificity, enhancing signal-to-noise ratios, and analysis of multiple epigenetic modifications are advantages of the method. Q^2^ ChIP can be performed in only one day [101]. Also, microChIP (μChIP) assay is another improved ChIP-based method, which is appropriate for up to nine parallel ChIPs of modified histone of 1,000 cells. The assay is applied to genome-wide studies and analysis of multiple epigenetic modifications, too [81, 102, 103]. On the other hand, the Fast-ChIP assay has improved the basic ChIP assay in two steps, and this improvement accelerates the protocol significantly and helps to detect different epigenetic factors [104, 105] in more than one site or even through the whole genome.

In the first step, for efficient antibody–protein binding, antibodies are incubated in an ultrasonic bath and the incubation time will decrease to 15 min. Secondly, elution of the ChIP complex, the reversal of cross-linking, and proteinase K digestion of bound proteins reduce the total time for the preparation of PCR-ready templates to 1 h (Table 1). To increase the scale of the ChIP assay, and simplify it, a microplate-based ChIP assay, Matrix-ChIP, has been developed in which antibody binding capacity is enhanced along with minimal sampling. Additionally, it is fast (one day) for the detection of both low and high-affinity proteins. The method is highly sensitive and automated (Table 1) (Fig. 4b) [81, 106].

### Site-specific analysis of histone modifications

During the past years, many studies have pointed to various roles of histone acetylation, and methylations as nuclear proteins [107]. Histones contribute to the formation of nucleosomes’ structure, and they are also subjects of post-translational modifications. Dynamic changes of chromatin structures are directly affected by post-translational modifications on amino-terminal tails of histones. Particular amino acids in histone tails undergo several post-translational modifications, including acetylation, phosphorylation, poly-ADP-ribosylation, ubiquitination, and methylation. These covalent modifications may change DNA-histone interaction that may lead to the regulation of various downstream cellular processes. Therefore, covalent histone modifications are essential for gene expression and regulation [107]. Histone modifications play significant roles in the regulation of DNA expression of many biological processes. Histone acetylation and methylation are two major players in gene activation and suppression at chromosomal domains. Single or a combination of histone acetylation regulates many cellular processes. Specific acetylation marks create distinct results singly or in combination. Interestingly, histone modifications are assessable by ChIP assay.

Technically, the site of interest on immune-precipitated-chromatin and also site-specific histone acetylation, and methylation could be analyzable on the genome. The ChIP assay is performed to identify a specific site and analyzes the amount and quality of precipitated chromatin (Fig. 4b). *MNase* digestion (*NchIP*) is particularly suitable for the preparation of input chromatin for site-specific histone modification analysis. The next steps perform the incubation of fragmented chromatin with antiserum against specific histone modification marks. Therefore, the percentage of precipitated chromatin is not the same as different histone modifications. The histone modification is abundantly present on chromosome precipitate, greatly more than that found in a small proportion of chromatin.

### *DNase I* hypersensitivity analysis of chromatin structure

In eukaryotes, chromatin has wrapped DNA, which forms the basic unit of the nucleosome. A nucleosome includes DNA that twice turns around four pairs of histone proteins linked with a DNA strand. Nucleosomes gather into higher-order structures as chromatin. Chromatin structures are deeply located in or near promoters and enhancers [13, 108]. Condensed DNA structures are usually inaccessible for TF binding, which prevents gene expression. Rapid progress in this field creates a passion for DNA hypersensitive analysis as a tool that identifies potential genomic transcription sites. DNA methylation refers to dideoxy cytosine (dc) methylation in CG pairs that form deoxy-methylcytosine (d^m^C). Methylcytosine binding proteins (such as MeCP2) bind to d^m^C and absorb chromatin complexes, including histone deacetylase. The complexes remove an acetyl group from histones and expand chromatin condensation in an inactivated state. Methylation on H3-K9 provides an HP1 binding site that triggers chromatin silencing. The technique commonly demonstrates chromatin acetylation and methylation consequences [13]. While the open configuration of DNA is exposed to TF binding and permits gene expression, condensed chromatin should remodel for TFs binding to their known sequence and convert into an open configuration to initiate the transcription [109]. This structure allows interaction between DNA and TFs and is an accessible structure for *DNase I* endonuclease.

Localized accessibility of *DNase I* into DNA and its digestion are the basis of the *DNase I* hypersensitivity assay, which performs limited digestion at inaccessible sites. Active transcription sites of DNA are the access configuration for TF binding. DNA-hypersensitive (DH) sites mostly map at or near known sequences for promoter-specific DNA binding proteins. This method has been very beneficial in the identification of many *cis*-regulatory elements such as promoters, insulators, and silencers [110].

DNA-hypersensitivity assay (DHS) is a powerful technique for analysis of the open structure of chromatin and genomic regulatory processes [108]. This method utilizes *DNase I* cleavage activity on hypersensitive sites, which is a fundamental property of chromatin structure in eukaryotes. Mapping of these regions determines many particular functions, including activation/inactivation of promoters, suppression/induction of genes, transcription silencing, structuring origin of replication, influencing recombination elements, and structural sites within or around telomerase and centromeres [111]. These sites of the genome are closely condensed and provide functional crosstalk with transcriptional regulatory machines and essentially TFs [112].

A general strategy (Fig. 4b) for DNA-hypersensitive analysis includes chromatin *DNase I* of limited treatment, which produces many hypersensitive fragments with variable lengths. To avoid under/over digestion, an optimized concentration, temperature, and duration of *DNase* treatment are necessary for each sample [13, 113]. The digestion product is purified, sequenced, and mapped to the genome. Finally, Southern blotting validation may be required. *DNase I* digestion is a good choice to compare the dynamics of chromatin structure between two or more conditions [13, 108].

Interestingly, the integration of this method with high-throughput methods (e.g., *DNase*-chip) can help to map genome-wide DNA-hypersensitive sites to epigenetic modifications [111]. *DNase I* hypersensitive sites have helped define important DNA sequences that have no distinct function [114]. Recently, DNA sequencing provides high-resolution genome-wide maps of DHS sites [108]. Mapping of DNA-hypersensitive sites or monitoring their kinetics of appearance demonstrates dynamic changes, in chromatin structure, around the specific genes that were transcriptionally verified, during the differentiation.

## Conclusion and future considerations

Investigating epigenetic modifications and translation of the results to the clinic is a critical challenge, especially in different cancers. Integration of epigenetic regulations with transcriptional changes can reveal the mechanism by which epigenetics is orchestrated with gene expression in response to intrinsic and extrinsic stimulations [115]. However, the information inferred from the epigenetic analysis does not provide sufficient data to understand mass and signal flow in biochemical pathways, but it is straightforward in the identification of causality of mutations and modifications in diseases. Moreover, the role of epigenetic regulations in the investigation of early life can help us to predict and prevent future risks of various diseases, which is a very important approach to personalized medicine [116]. Therefore, the identification of epigenetic biomarkers and targeted epigenetic therapy of diseases including DNA methylation and histone modifications [117] should be strengthened urgently, which needs good knowledge of the relative methodologies.

## Data Availability

Please contact the corresponding author for data requests.

## Abbreviations

DNMT: DNA methyltransferase
TET: Ten-eleven Translocation
MBD: Methyl-cytosine binding domain
SAM: S-adenosyl methionine
CGIs: CpG islands
CHARM: Comprehensive high-throughput arrays for relative methylation
DMH: Differential methylation hybridization
HELP: HpaII tiny fragment enrichment by ligation-mediated PCR
MBD: Methyl-CpG binding domain
MCAM: Methylated CpG island amplification microarray
5-meC: 5-Methyl cytosine
MeDIP: Methylated DNA immunoprecipitation
MIRA: Methylated-CpG island recovery assay
MMASS: Microarray-based methylation assessment of single samples
MSCC: Methyl-sensitive cut counting
MSO: Methylation-specific oligonucleotides
PMAD: Promoter-associated methylated DNA amplification DNA chip
RRBS: Reduced representation bisulfite sequencing
NGS: Next-generation sequencing
SNuPE: Methylation-sensitive single nucleotide primer extension
BSP: Bisulfite sequencing PCR
COBRA: Combined bisulfite restriction analysis
MALDI-TOF: Matrix-assisted laser desorption ionization time-of-flight
RLGS: Restriction landmark genomic scanning
LUMA: Luminometric methylation assay
MS-MLPA: Methylation-specific multiplex ligation-dependent probe amplification
MS-AFLP: Methylation-sensitive amplified fragment length polymorphism
