# Correction: Laboratory methods to decipher epigenetic signatures: a comparative review

**DOI:** 10.1186/s11658-022-00387-9

**Published:** 2022-09-21

**Authors:** Raheleh Halabian, Valizadeh Arshad, Ali Ahmadi, Pardis Saeedi, Sadegh Azimzadeh Jamalkandi, Mohammad Reza Alivand

**Affiliations:** 1 1Applied Microbiology Research Center, Systems Biology and Poisonings Institute, Baqiyatallah University of Medical Sciences, Tehran, Iran; 2 Department of Stem Cell and Developmental Biology, Cell Science Research Center, Royan Institute For Stem Cell Biology and Technology, ACECR, Tehran, Iran; 3 3Molecular Biology Research Center, Systems Biology and Poisonings Institute, Baqiyatallah University of Medical Sciences, Tehran, Iran; 4 Chemical Injuries Research Center, Systems Biology and Poisonings Institute, Baqiyatallah University of Medical Sciences, Mollasadra Ave, 14359-16471 Tehran, Iran; 5 Department of Medical Genetics, Faculty of Medicine, Tabriz University of Medical Sciences, Tabriz, Iran

Correction: Cellular & Molecular Biology Letters (2021) 26:46 10.1186/s11658-021-00290-9.

Following publication of the original article [1], we have been informed that the affiliation of the author Raheleh Halabian has been incorrectly assigned.

The affiliation group has been updated above and the original article has been corrected.

## References

[1] Halabian, et al. Cellular & Molecular Biology Letters (2021) 26:46 10.1186/s11658-021-00290-9. 10.1186/s11658-021-00290-9 PMC8582164 34763654

